# Training and Preparation Methods Relevant to Elite 5000 m Track Performance: A Systematic Review

**DOI:** 10.3390/sports14090397

**Published:** 2026-09-07

**Authors:** Clinton Deon Swanepoel, Charl Jacobus Roux, Heather Morris-Eyton

**Affiliations:** Department of Sport and Movement Studies, Faculty of Health Sciences, University of Johannesburg, Johannesburg 2028, South Africaheatherm@uj.ac.za (H.M.-E.)

**Keywords:** pacing behavior, altitude adaptation, running economy, tapering, warm-up, energy availability, advanced footwear technology, endurance physiology

## Abstract

Elite 5000 m track performance depends on the integration of endurance training, race-specific speed, tactical decision-making, technology, physiological characteristics, and individualized athlete support. This systematic review synthesized peer-reviewed evidence available as open-access full texts that was either specific to the 5000 m event or explicitly applicable to elite 5000 m preparation. Searches of SPORTDiscus, ProQuest Central, Scopus, and PsycINFO were completed in February 2025 and updated on 17 August 2026, with reporting guided by PRISMA 2020 and PRISMA-S and narrative synthesis structured with reference to SWiM. Following duplicate removal, 1439 records were screened; 430 reports were sought, 39 were not retrieved, 391 were assessed, and 11 publications met the deliberately narrow event-relevance criteria. The update search identified 11 records; three duplicates were removed, eight unique records were screened, and no additional eligible publication was identified. Methodological and reporting quality were appraised using QuADS. Evidence was considered separately across warm-up, training-intensity distribution, tapering, altitude exposure/training responses, pacing/tactical behavior, footwear technology, physiological profiling/performance determinants, and nutrition. The most direct event-specific evidence comprised a 5000 m warm-up trial in trained runners and observational championship pacing analyses, whereas most other evidence was indirect to exclusive elite 5000 m specialists. Confidence was low or very low across components. The pragmatic open-access retrieval restriction, small evidence base, methodological heterogeneity, restricted database coverage, and indirectness limit certainty and generalizability. The findings should therefore be interpreted as conditional considerations rather than prescriptive recommendations.

## 1. Introduction

The 5000 m is classified by World Athletics as a long-distance track event, but its 12.5-lap competitive demands combine sustained aerobic power with race-specific speed, anaerobic support, tactical positioning, repeated pace changes, and a decisive finishing phase [1,2,3,4]. This combination distinguishes the event from both evenly paced endurance time trials and shorter middle-distance races. Consequently, preparation must address not only aerobic development and running economy, but also the capacity to tolerate surges, maintain position, and produce high speed under fatigue.

At international level, very small performance differences can determine qualification, finalist status, or medal outcomes. Training-intensity distribution, tapering, altitude exposure, warm-up design, pacing behavior, footwear technology, body-composition management, hydration, and energy availability may all influence preparation [5,6,7,8,9,10,11]. However, these factors have usually been studied separately, and much of the available literature combines 5000 m athletes with broader middle- or long-distance cohorts.

The term elite is frequently used inconsistently in sport science. For this review, elite status was operationalized using the logic of the participant-classification framework proposed by McKay et al. [12]: athletes were required to be described as international, world-class, senior national representatives, major-championship competitors, or equivalent Tier 4–5 performers. Because some event-specific studies used trained runners rather than confirmed Tier 4–5 athletes, the review distinguished direct 5000 m evidence from evidence that was indirect with respect to participant caliber. This distinction is important because a direct 5000 m outcome does not automatically establish direct applicability to world-class athletes.

The review addressed the following question: What training and preparation methods have been reported in evidence that is directly specific to the 5000 m event or explicitly relevant to elite adult 5000 m track performance? The purpose was not to infer a universal preparation model from a small literature base, but to map the available evidence, assess its methodological quality and directness, identify areas of convergence and uncertainty, and provide appropriately qualified implications for coaches, athletes, and sport scientists.

Existing reviews relevant to this literature generally address single preparation areas or broader middle- and long-distance populations, including nutrition, physiology, training organization, and altitude [5,6,8,13]. The present review differs by retaining a 5000 m event focus, operationalizing elite participant caliber, separating direct from indirect evidence, distinguishing primary empirical from secondary/conceptual evidence, and considering confidence separately across preparation components. This approach is intended to clarify both what is supported specifically for the 5000 m and where interpretation depends on extrapolation.

## 2. Materials and Methods

### 2.1. Review Design, Protocol, and Reporting Framework

This systematic review was designed to identify, appraise, and synthesize evidence relevant to elite 5000 m training and preparation. The review was managed in Covidence systematic review software (Veritas Health Innovation, Melbourne, Australia) [14]. Covidence is a web-based platform that does not use fixed version numbers; it was accessed during the original review in February 2025 and during the update on 17 August 2026. Reporting followed PRISMA 2020 (Table S1) [15,16]. Search reporting was additionally informed by PRISMA-S [17], and the synthesis without meta-analysis was structured with reference to SWiM [18]. Because the included evidence was heterogeneous in design and directness, the findings are presented using an event-focused evidence-mapping approach within the systematic review rather than as a pooled intervention-effect estimate. No protocol was prospectively registered. The review question, eligibility criteria, and principal extraction categories were established before screening; nevertheless, the absence of prospective registration is acknowledged as a limitation because it prevents independent verification of whether all methodological decisions remained unchanged.

### 2.2. Eligibility Framework (PICOS)

Eligibility criteria were structured using the PICOS framework [19]. Because the review addressed a heterogeneous preparation question rather than a single intervention effect, the comparator element included descriptive and comparative evidence. Population eligibility prioritized adult Tier 4–5 5000 m athletes. Mixed elite middle- or long-distance cohorts were eligible only when the study reported extractable 5000 m-specific findings or when the studied event range explicitly included the 5000 m and the preparation/performance construct had a direct application to 5000 m track performance. Broader endurance evidence without an explicit 5000 m-specific finding or stated 5000 m event-range link was excluded from the formal evidence set. Event-specific studies using trained but not confirmed elite runners were retained as indirect evidence and were not treated as equivalent to evidence from elite 5000 m specialists (Table 1).

### 2.3. Information Sources and Search Strategy

The original search was completed in February 2025 in SPORTDiscus (EBSCOhost), ProQuest Central, Scopus, and PsycINFO (EBSCOhost). These databases were selected to provide complementary sport/exercise, coaching, psychology, and multidisciplinary coverage; SPORTDiscus supplied sport-specific indexing, while Scopus and ProQuest Central broadened multidisciplinary coverage. A dedicated biomedical database such as MEDLINE or Embase was not searched, which may have reduced retrieval completeness for some physiology, nutrition, sports-medicine, and altitude literature and is acknowledged as a limitation. The search used three concept blocks: event (“5000 meter track” OR “5000 m track” OR “middle distance running” OR “long distance track running”), population (“elite runner*” OR elite OR international OR “world class”), and preparation (train* OR prepar* OR coach* OR pacing OR taper* OR altitude OR nutrition OR footwear), combined with AND between blocks and OR within blocks. No publication-date restriction was applied to the original search. Full database-specific syntax, platforms, searched fields, filters, truncation rules, and original retrieval counts are reported in Supplementary Table S2. Because the exact original calendar day and line-by-line database histories were not retained, the historical syntax is transparently reported as a reconstruction from the archived search concepts rather than as an exact exported history. The same core search concepts were rerun on 17 August 2026 over an overlapping 2025–2026 date window. The update identified 11 records (SPORTDiscus *n* = 5; ProQuest Central *n* = 0; Scopus *n* = 6; PsycINFO *n* = 0); 3 duplicates were removed; 8 unique records were screened; and no additional eligible publication was identified. Records were managed in Covidence [14].

### 2.4. Eligibility Criteria

Studies were eligible when they were peer-reviewed, written in English, involved adults, and either (a) examined elite 5000 m athletes directly, (b) reported extractable 5000 m-specific findings within an elite mixed-distance cohort, or (c) evaluated a preparation/performance construct meeting the operational event-relevance definition described above. Open-access full-text availability was applied as a pragmatic retrieval restriction rather than a scientific-quality criterion. Reports were excluded when they focused only on youth, recreational, beginner, or amateur populations; other events without extractable or explicit 5000 m applicability; non-track endurance activities; clinical or epidemiological outcomes unrelated to preparation; non-peer-reviewed sources; or when open-access full text was not retrievable. No systematic attempts were made to obtain subscription-only reports through institutional access, repositories, or author contact. This availability restriction may therefore have excluded scientifically relevant evidence and is acknowledged in the Abstract and Discussion. The inclusion and exclusion criteria are summarized in Table 2.

### 2.5. Study Selection

**Figure 1:**
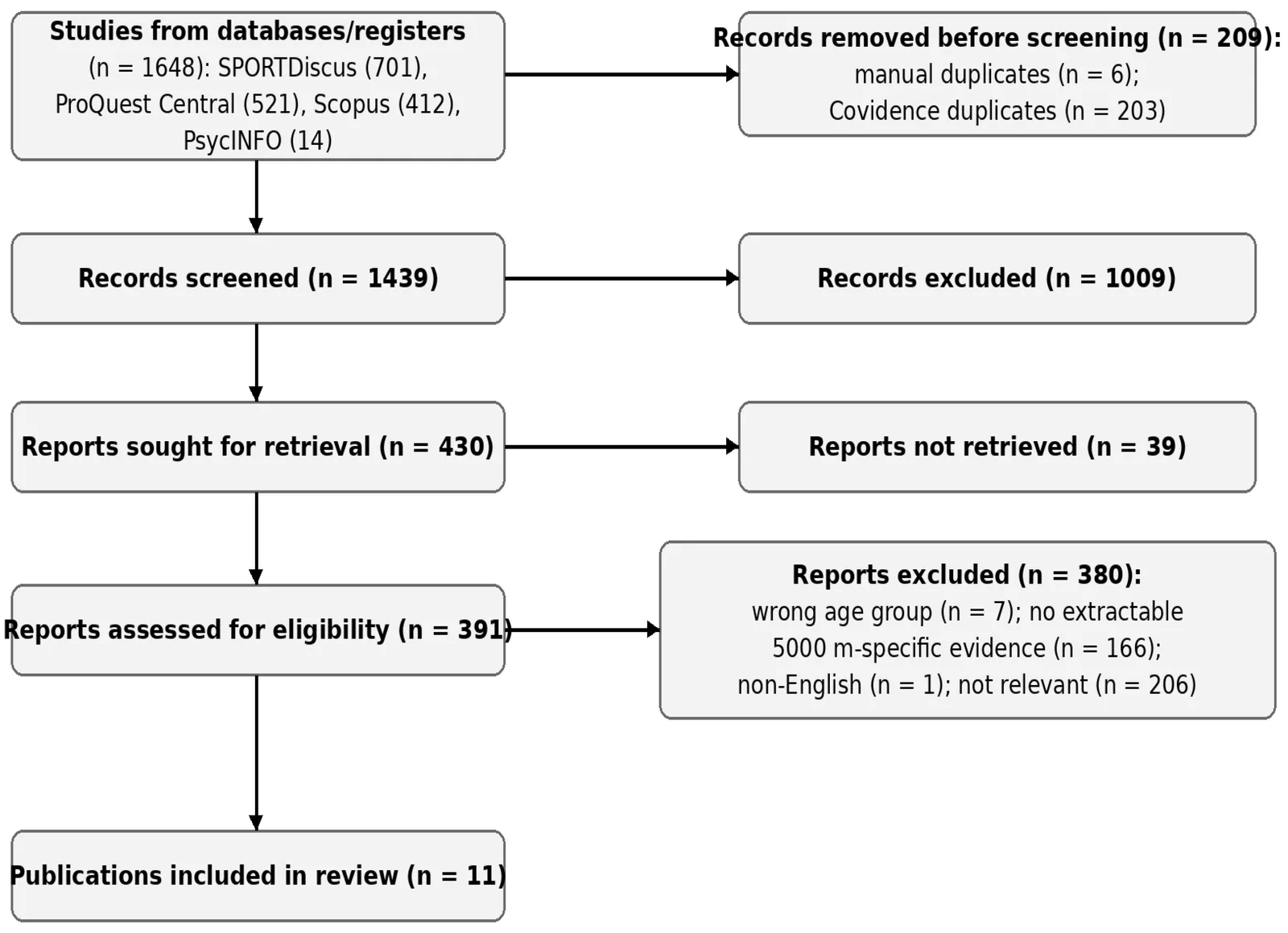
PRISMA 2020 flow diagram for study identification and selection.

The original search identified 1648 records. After removal of 209 duplicates (6 manually and 203 in Covidence), 1439 records underwent title and abstract screening. A total of 1009 records were excluded and 430 reports were sought for retrieval. Thirty-nine reports were not retrieved because the open-access full text was unavailable; 391 reports were therefore assessed for eligibility. Of these, 380 were excluded: wrong age group (*n* = 7), no extractable 5000 m-specific evidence (*n* = 166), non-English publication (*n* = 1), and not relevant to the preparation question (*n* = 206). The latter category included reports on recreational participation, non-preparation clinical or epidemiological outcomes, unrelated running events, and physiological descriptions without an extractable link to training or preparation. Eleven publications met the event-relevance criteria and were included (Figure 1). The 17 August 2026 update search identified no additional eligible publication, so the formal evidence set remained unchanged.

### 2.6. Data Extraction and Synthesis

Data were extracted into a structured matrix containing study design, participant caliber, event directness, preparation component, objective findings, and review interpretation. Title/abstract screening and full-text eligibility assessment were completed by a minimum of two reviewers in Covidence, and disagreements were resolved by consensus before a final decision was recorded. Data extraction was likewise completed and agreed by at least two reviewers. Where reported and relevant, the final extraction included sample size, sex, age, competitive level, event specialization, performance information, intervention or exposure, quantitative outcomes, measures of variability, and inferential statistics. Meta-analysis was not appropriate because the included evidence differed substantially in study design, populations, interventions or exposures, and outcomes. In accordance with the SWiM-informed narrative approach [18], publications were grouped by preparation component; direction and magnitude were taken from the reported study results rather than vote-counted, and methodological quality, consistency, event/participant directness, evidence volume, and publication type were considered when interpreting each component.

### 2.7. Quality Appraisal

The methodological and reporting quality of each included publication was assessed using QuADS [20]. QuADS was retained because the evidence set included experimental, observational, descriptive, review-based, and conceptual publications and therefore required a common appraisal framework. Its 13 criteria assess conceptual grounding, research setting, sampling, data collection, analysis, stakeholder involvement, and reporting, with scores from 0 to 3 and a maximum of 39. The lead author completed the appraisal and discussed uncertain scores with the co-authors. The prespecified percentage bands (>75%, 50–74%, <50%) are reported descriptively only and are not interpreted as validated levels of methodological validity or as design-specific risk-of-bias judgments. Item-level scores and criteria are provided in Supplementary Table S3. QuADS does not replace design-specific risk-of-bias assessment, and this limitation was considered in the narrative confidence judgments.

### 2.8. Reporting-Bias Assessment

Formal funnel-plot or regression-based assessment of small-study effects was not appropriate because no preparation domain contained at least 10 sufficiently comparable studies and no pooled effect estimates were calculated. Potential reporting bias was therefore considered narratively through selective outcome reporting, publication type, full-text availability, and the absence of unpublished evidence. The inability to quantify publication bias reduces confidence in the synthesis.

### 2.9. Structured Confidence Assessment

Confidence was assessed separately for each preparation component using a structured narrative framework informed by, but not equivalent to, GRADE concepts [19]. For each component, the assessment considered the initial evidence position, methodological limitations (including QuADS and study design), inconsistency, indirectness to elite 5000 m athletes, imprecision/evidence volume, and potential publication bias. Because no pooled intervention effects were estimated and the evidence included heterogeneous designs, these ratings are narrative confidence judgments rather than a formal GRADE assessment. The component-level rationale is reported in Supplementary Table S4.

## 3. Results

### 3.1. Quality of Included Studies

Using the prespecified descriptive QuADS bands, four publications scored above 75%, six scored between 50% and 74%, and one scored below 50%; these bands are descriptive and are not formal validity ratings. Aragon et al. [4] obtained the highest corrected total (36/39; 92.3%), whereas Jones and Whipp [7] obtained the lowest score (13/39; 33.3%). Confidence was not determined by the total score alone and was reduced where evidence relied on broader cohorts, secondary or conceptual publications, indirectness, or limited evidence volume (Table 3).

### 3.2. Overview of Included Studies

The 11 included publications each provided a 5000 m-specific outcome or an explicitly relevant preparation construct, but directness and publication type varied. Six were primary empirical publications (experimental or observational) and five were secondary or conceptual publications; these were not treated as equivalent contributions to causal evidence. The most direct event evidence came from a 5000 m warm-up trial and championship pacing analyses [3,4,21], whereas other components relied mainly on mixed-distance cohorts or secondary evidence [5,6,7,8,9,10,11,22]. Among the six primary empirical publications, two were male-only and four included both sexes; no female-only elite 5000 m intervention study was identified, and sex-specific results were limited. Table 4 reports study type, participant characteristics, quantitative findings where available, and event/participant directness.

### 3.3. Running-Specific Structured Training Programs

Running-specific preparation was represented by five heterogeneous publications [8,9,11,21,22]. Alves et al. [21] directly measured 5000 m performance in 13 trained male endurance runners and reported a 6.4 s (0.5%) faster time after the high-intensity warm-up than after the low-intensity warm-up (1141.4 ± 110.4 vs. 1147.8 ± 111.0 s; *p* = 0.03; Hedges *g* = 0.66); because the participants were not confirmed Tier 4–5 athletes, the finding is event-specific but indirect to elite caliber. Kenneally et al. [22] and Tjelta [8] described training-intensity distribution in world-class or international distance runners. Kenneally et al. showed that race-pace classification produced a predominantly pyramidal distribution, whereas physiological-zone classification changed the apparent distribution and showed greater individual variability. These publications do not establish a single optimal distribution for all 5000 m athletes.

Tapering and altitude evidence were similarly conditional. Spilsbury et al. [11] showed that taper practices varied by event and habitual training, which supports individualization but does not identify one superior taper. Sharma et al. [9] assessed training responses at 2100 m in elite middle-distance runners and found reduced running speed and increased perceived exertion at higher intensities. This finding is therefore described as a training response during altitude exposure, not as evidence that an altitude-training strategy improves or impairs 5000 m competition performance. Across these components, the interventions/exposures and outcomes were too heterogeneous for a pooled effect estimate.

### 3.4. Tactical Techniques and Technology

The most event-specific tactical evidence came from major-championship analyses. Thiel et al. [3] documented substantial within-race speed variation and strong finishing phases in Olympic track races, while Aragon et al. [4] identified changes in pace, positioning, and sprint initiation zones in winning 1500 m and 5000 m finalists. The studies converge in showing that championship 5000 m performance is not adequately represented by an even-pace model. Nevertheless, both were observational analyses of successful races and cannot determine whether rehearsing a particular tactical pattern causes improved performance.

Footwear evidence was less event-specific. Healey et al. [10] described mechanisms through which advanced track spikes may reduce energy cost, but the magnitude of benefit is athlete-, speed-, and model-dependent. Because this was secondary/mechanistic evidence rather than a direct elite 5000 m outcome study, it was treated as indirect within the formal evidence set.

### 3.5. Physiological Profiling and Performance Determinants

Physiological evidence was represented by one broad review of Kenyan runners [6] and was therefore indirect to elite 5000 m preparation. The evidence supports a multifactorial explanation involving maximal oxygen uptake, fractional utilization, running economy, training environment, and lifestyle rather than a single genetic or physiological determinant. Because no included study prospectively tested an individualized physiological intervention in elite 5000 m specialists, these characteristics should be interpreted as profiling considerations rather than proven preparation methods.

### 3.6. Nutritional Needs

Nutrition evidence was also indirect. Stellingwerff et al. [5] addressed contemporary nutrition for middle-distance runners and emphasized carbohydrate availability, protein intake, hydration, energy availability, and strategic rather than chronic body-composition manipulation. These principles are physiologically relevant to 5000 m preparation, but the review identified no event-specific controlled study in elite 5000 m athletes. The confidence assigned to this theme was therefore very low, despite the practical importance of preventing low energy availability and relative energy deficiency in sport.

### 3.7. Structured Confidence in the Evidence

The synthesis identified plausible preparation considerations rather than a validated, event-specific prescription. To avoid combining substantially different questions, confidence was assessed separately for warm-up, training-intensity distribution, tapering, altitude exposure/training responses, pacing/tactical behavior, footwear technology, physiological profiling/performance determinants, and nutrition. Confidence was low for warm-up, training-intensity distribution, and pacing/tactical behavior, and very low for tapering, altitude exposure/training responses, footwear technology, physiological profiling/performance determinants, and nutrition (Table 5).

## 4. Discussion

This review intentionally applied narrow event-relevance criteria and consequently included only 11 publications. The small number should not be interpreted as evidence that research on endurance preparation is scarce in general; it reflects the interaction between the event-focused eligibility criteria and the review’s database, language, access, and retrieval restrictions. The principal scientific contribution is therefore an event-focused evidence map within a systematic review that distinguishes direct evidence from extrapolation and identifies where common coaching practices rest on limited event-specific research.

Methodological strengths include the event-focused eligibility framework, the objective participant-caliber definition, dual-reviewer screening and data extraction, explicit separation of direct and indirect evidence, transparent classification of publication type, integration of QuADS into interpretation rather than exclusion, and a component-level narrative confidence assessment.

The training studies were directionally compatible with individualized preparation but did not support a universal program. The direct 5000 m warm-up trial favored a high-intensity protocol [21], consistent with broader evidence that warm-up structure can influence subsequent performance [23]. The broader training-distribution studies emphasized the interaction between high overall volume, predominantly low-intensity work, and smaller doses of race-relevant intensity [8,22], while wider endurance literature similarly demonstrates the importance of how training intensity is distributed [24]. The apparent agreement is limited because the studies addressed different questions and used different athlete classifications. Taper findings further suggest that the reduction in volume and maintenance of intensity should be interpreted relative to the athlete’s habitual training rather than applied as a fixed formula [11].

Altitude evidence illustrates the importance of separating adaptation goals from session quality. Reduced high-intensity speed and elevated perceived exertion at 2100 m [9] are relevant when race-pace work is prescribed at altitude, while the broader literature suggests that hematological or performance responses vary with exposure model, timing, and individual responsiveness [13,25]. The included evidence therefore supports monitoring and individualized periodization, but it does not establish a superior altitude strategy for elite 5000 m runners.

Tactical evidence was comparatively direct and internally consistent. Championship analyses show repeated microvariations in speed, positional changes, and decisive finishing phases [3,4]. These findings justify including surge tolerance and finishing speed in race-specific preparation; however, they do not prove that one location for a surge or sprint is optimal across races. Tactical choices are constrained by competitors, race context, and the athlete’s available physiological reserve [7,26].

Advanced footwear technology is a credible source of marginal gain, but the evidence base has evolved rapidly. Mechanical explanations relevant to running economy [10,27] are now supported by experimental and performance studies showing benefits for some, but not all, advanced-spike models [28,29,30]. Athlete-specific responses, running speed, shoe geometry, comfort, and familiarization may modify the effect. For practice, the defensible recommendation is controlled individual testing in race-relevant conditions rather than selection based solely on the presence of a carbon plate.

Physiological profiling and nutrition are central to athlete management, but they were the least directly supported themes in this event-specific review. Running economy, maximal oxygen uptake, and fractional utilization describe important performance characteristics [6,31], while adequate carbohydrate availability and fluid replacement support training, recovery, health, and performance [5,32,33,34]. The evidence does not demonstrate that a single physiological profile or nutrition protocol produces elite 5000 m success. These domains should therefore inform individualized assessment and multidisciplinary support, not deterministic athlete selection or prescriptive targets.

### 4.1. Practical Implications

For coaches and athletes, the evidence supports a cautious, individualized decision framework. A high-intensity warm-up may warrant individualized evaluation before competition; training distribution may be considered using both race-pace and physiological-zone information; tapering and training at altitude may be adjusted relative to habitual load and observed response; descriptive pacing findings may inform, but do not prove the effectiveness of, tactical rehearsal; footwear may be individually tested within the rules and under race-relevant conditions; and nutrition support should prioritize energy availability, recovery, and health. These are conditional applications of limited evidence and include practice-based inference where direct causal evidence is unavailable.

### 4.2. Limitations

This review has important limitations. First, only 11 publications met the narrow event-relevance criteria, and several involved mixed middle- or long-distance cohorts, trained but non-Tier 4–5 participants, narrative reviews, or conceptual evidence rather than exclusive elite 5000 m samples. Second, the operational event-relevance criterion necessarily required reviewer judgment, although title/abstract and full-text eligibility decisions were completed by a minimum of two reviewers with consensus. Third, no protocol was prospectively registered. Fourth, the search was restricted to English-language, peer-reviewed literature retrievable as open-access full texts; 39 reports were not retrieved because open-access full texts were unavailable, and no systematic institutional-library, repository, or author-contact retrieval process was undertaken. This pragmatic restriction may have excluded scientifically relevant subscription-based evidence. Fifth, the database set did not include a dedicated biomedical database such as MEDLINE or Embase, and the exact original search day and line-by-line histories were not retained; the historical syntax is therefore a transparent reconstruction from archived concepts. Sixth, the evidence base mixed primary, secondary, and conceptual publication types. Secondary/conceptual sources were separated from primary empirical evidence and were not treated as independent effect estimates, but overlap in the underlying literature remains possible. Seventh, sex-specific applicability is limited: the primary evidence included male-only and mixed-sex samples, with no female-only elite 5000 m intervention study identified. Eighth, diverse designs and outcomes precluded meta-analysis, funnel-plot assessment, and statistical evaluation of small-study effects. Finally, QuADS provided a common methodological/reporting appraisal but did not replace design-specific risk-of-bias tools. These limitations, together with indirectness and limited evidence volume, reduce confidence in the conclusions.

### 4.3. Future Directions

Future research should prioritize prospective longitudinal studies of Tier 4–5 5000 m specialists, with clear reporting of sex, personal best, ranking, championship level, training age, and event specialization. Priorities include comparative warm-up trials in elite athletes; season-long training-load and race-pace studies; event-specific taper experiments; altitude periodization linked to return-to-sea-level timing; integrated physiology–tactics studies that examine surge response and finishing speed; female-specific research; individualized advanced-spike response testing; and nutrition studies that connect energy availability with training quality, injury, health, and competition outcomes. Standardized participant classification and reporting would materially improve future synthesis.

## 5. Conclusions

The available evidence suggests that elite 5000 m preparation is multifactorial and should be individualized, but the certainty of event-specific evidence is limited. The most direct findings comprise an experimental 5000 m warm-up result in trained male runners and descriptive championship pacing observations in elite competition; neither establishes a universally effective preparation method. Training-intensity distribution, tapering, altitude exposure/training responses, footwear, physiological profiling/performance determinants, and nutrition remain relevant considerations, but much of the evidence is heterogeneous, secondary, or extrapolated from mixed-distance cohorts. Practitioners should therefore use the findings as conditional, evidence-informed considerations, monitor individual response, and avoid treating any single method as universally effective. The review identifies plausible components and clear evidence gaps rather than a definitive elite 5000 m preparation model.

## Figures and Tables

**Table 1:** PICOS framework.

Element	Description
Population (P)	Adult elite/international or world-class 5000 m athletes (Tier 4–5), or mixed elite cohorts with extractable 5000 m-specific evidence. Event-specific trained-runner studies were retained only as indirect evidence.
Intervention (I)	Training and preparation strategies relevant to 5000 m performance, including training structure, warm-up, tapering, altitude, tactics, technology, physiology, and nutrition.
Comparator (C)	Alternative preparation approaches, within-athlete conditions, athlete groups, race phases, or descriptive evidence where no formal comparator was used.
Outcome (O)	5000 m performance, race behavior, training organization, physiological or perceptual response, running economy, and preparation-related nutrition outcomes.
Study design (S)	Peer-reviewed quantitative, qualitative, mixed-method, observational, experimental, or review evidence meeting the event-relevance criteria.

**Table 2:** Inclusion and exclusion criteria.

Category	Inclusion Criteria	Exclusion Criteria
Language	English-language publication.	Non-English publication.
Peer review	Peer-reviewed journal article.	Gray literature, books, theses, dissertations, conference abstracts, or non-peer-reviewed sources.
Access	Open-access full text available.	Abstract-only record, subscription-only article, or open-access full text not obtainable.
Event focus	Direct 5000 m evidence or an explicitly relevant strategy with extractable 5000 m applicability.	Other event or discipline without extractable 5000 m-specific evidence.
Participant caliber	Adult Tier 4–5/international/world-class athletes; mixed elite cohorts with extractable 5000 m evidence.	Youth, recreational, beginner, amateur, or non-elite cohorts without direct 5000 m outcome data.
Preparation relevance	Training, warm-up, tapering, altitude, tactics, technology, physiology, or nutrition linked to preparation/performance.	Clinical, epidemiological, participation, or descriptive outcomes without a preparation link.

**Table 3:** Quality assessment using the Quality Appraisal for Diverse Studies (QuADS) tool. * Percentage categories are descriptive only and are not validated levels of methodological validity.

Study	1	2	3	4	5	6	7	8	9	10	11	12	13	Total	%	Descriptive Category *
Alves et al. (2023) [21]	2	3	3	3	2	2	3	3	1	2	3	1	2	30	76.9%	High
Aragon et al. (2016) [4]	2	3	3	3	3	3	3	3	3	3	3	3	1	36	92.3%	High
Healey et al. (2022) [10]	1	1	3	2	2	2	2	1	2	2	2	1	1	22	56.4%	Medium
Tjelta (2016) [8]	2	2	2	3	3	2	3	3	2	3	2	2	0	29	74.4%	Medium
Jones and Whipp (2002) [7]	0	0	2	1	2	0	1	2	2	1	2	0	0	13	33.3%	Low
Kenneally et al. (2021) [22]	1	3	3	3	3	3	3	3	3	2	3	2	0	32	82.1%	High
Larsen and Sheel (2015) [6]	1	2	2	2	3	2	2	3	3	2	2	3	1	28	71.8%	Medium
Sharma et al. (2017) [9]	0	3	3	3	3	3	3	2	2	2	2	0	0	26	66.7%	Medium
Spilsbury et al. (2015) [11]	1	3	3	3	2	2	3	2	2	1	2	2	1	27	69.2%	Medium
Stellingwerff et al. (2019) [5]	2	3	3	3	3	2	2	3	2	3	3	1	1	31	79.5%	High
Thiel et al. (2012) [3]	1	2	3	3	3	3	2	2	2	2	3	1	1	28	71.8%	Medium

**Table 4:** Comprehensive overview of included study results.

Study/Design	Population, Caliber and Event Directness	Intervention/Exposure and Main Finding	Review Interpretation and Caveat
Alves et al. [21]—randomized crossover	*n* = 13 men; 34 ± 10 y; trained endurance runners; direct 5000 m time trial; Tier 4–5 caliber not confirmed.	Low- vs. high-intensity warm-up. HIWU included 1 × 500 m at 70% plus 3 × 250 m at 100%. 5000 m time: 1141.4 ± 110.4 vs. 1147.8 ± 111.0 s; 6.4 s (0.5%) faster after HIWU; *p* = 0.03; Hedges *g* = 0.66.	Event direct but participant caliber indirect. A high-intensity warm-up may warrant individual trialing; one study only.
Aragon et al. [4]—observational tactical analysis	Winning male athletes in major 1500 m and 5000 m finals; world/Olympic/European championship level; age and PB/ranking not reported in the extracted data.	Systematic observation/T-pattern analysis. Winning 5000 m performances showed identifiable pace changes, positioning actions and sprint initiation zones.	Elite and event-specific observational evidence; describes successful race behavior but does not establish a causal tactical-training effect.
Healey et al. [10]—narrative/mechanistic review	Track-running literature; no single participant sample; not exclusive to elite 5000 m athletes.	Reviewed mechanisms by which advanced track spikes may reduce mechanical energy loss/running economy; effect varies by model, speed, biomechanics and athlete response.	Secondary/mechanistic evidence; indirect to an elite 5000 m outcome.
Tjelta [8]—narrative review	International-level distance runners across events; no single study sample/age/PB applicable.	Reviewed high-volume training with varied intensity distributions in successful distance runners.	Secondary training evidence; indirect to an exclusive 5000 m cohort and not an independent intervention effect.
Jones and Whipp [7]—conceptual paper	Middle-distance bioenergetics; no empirical participant sample.	Conceptual model in which finite energetic resources constrain pacing and tactical distribution of effort.	Mechanistic rationale only; conceptual and indirect.
Kenneally et al. [22]—longitudinal observational	*n* = 7 world-class middle-/long-distance runners; mixed sex; event groups included 5000–10,000 m; age/PB not reported in the extracted data.	Fifty weeks of training. Race-pace classification showed a predominantly pyramidal distribution, whereas physiological-zone classification produced different Z2/Z3 distributions and greater individual variability.	Elite caliber but mixed events. Race-pace and physiological-zone classifications should not be treated as interchangeable.
Larsen and Sheel [6]—narrative review	Elite Kenyan distance runners; broad review; no single participant sample/age/PB applicable.	Reviewed VO_2_max, fractional utilization, running economy, training environment and lifestyle characteristics.	Secondary physiological evidence; informs profiling/performance determinants rather than proving a preparation intervention.
Sharma et al. [9]—observational altitude cohort	*n* = 19 (13 men, 6 women); 25 ± 5 y; elite 800–5000 m runners; VO_2_max 71 ± 5 mL/kg/min; PB/ranking not reported in the extracted data.	Altitude group trained 4–5 weeks while living at 2100 m and training at 1400–2700 m; control trained at sea level. Running speed decreased and session RPE increased at higher intensities at altitude.	Evidence of training response during altitude exposure, not evidence that an altitude-training strategy improves 5000 m competition performance.
Spilsbury et al. [11]—survey/observational	*n* = 37 (21 men, 16 women); 26 ± 5 y; senior British national/international/Olympic runners across events from 800 m to the marathon; small 5000 m subgroup.	Surveyed habitual training and pre-competition taper. Taper duration and reductions in continuous/interval volume varied by event and habitual training.	Elite caliber but mixed events; supports individualization, not one superior 5000 m taper.
Stellingwerff et al. [5]—narrative review	Middle-distance runners; review evidence; no single sample/age/PB applicable.	Reviewed carbohydrate/protein availability, hydration, energy availability and strategic body-composition management.	Secondary nutrition/health-support evidence; no event-specific controlled elite 5000 m study.
Thiel et al. [3]—observational race analysis	Olympic finalists; men and women; 800–10,000 m events including 5000 m; age/PB/ranking not reported in the extracted data.	Olympic race analysis showed repeated within-race speed variation and strong end spurts rather than world-record pacing patterns.	Elite and 5000 m inclusive observational evidence; descriptive rather than causal.

**Table 5:** Structured narrative confidence in evidence by preparation component.

Preparation Component	Evidence Basis	Basis for Confidence Judgment	Confidence	Interpretation
Warm-up	1 randomized crossover 5000 m trial	Direct event outcome but *n* = 13 trained men; Tier 4–5 caliber not confirmed; single study; limited precision.	Low	High-intensity warm-up may warrant individualized trialing; elite-specific effect remains uncertain.
Training-intensity distribution	1 world-class observational study + 1 review	Mixed events; classification method changes interpretation; no causal 5000 m intervention trial.	Low	Race-pace and physiological-zone definitions should be explicit; no universal distribution is established.
Tapering	1 elite survey/observational study	Mixed events; small 5000 m subgroup; no event-specific intervention.	Very low	Tapering may be individualized relative to habitual training; 5000 m-specific effect is uncertain.
Altitude exposure/training responses	1 elite observational cohort	800–5000 m mixed cohort; training-response outcome at altitude, not post-altitude 5000 m performance.	Very low	Training pace/load may require adjustment at altitude; effectiveness of an altitude strategy remains uncertain.
Pacing/tactical behavior	2 elite observational analyses + 1 conceptual paper	Event-specific descriptive evidence but no causal tactical-training trial.	Low	Variable championship pacing is well described; causal benefits of rehearsing a specific tactical pattern are unproven.
Footwear technology	1 secondary/mechanistic review	No direct included elite 5000 m outcome; model-, speed- and athlete-dependent response.	Very low	Individual testing may be considered; event-specific performance effect remains uncertain.
Physiological profiling/performance determinants	1 narrative review	Secondary evidence; broad cohort; not a preparation intervention.	Very low	Useful for profiling and interpretation, not a proven preparation method.
Nutrition	1 narrative review	Secondary evidence; no controlled elite 5000 m study.	Very low	Relevant to health and training support; event-specific performance effects remain uncertain.

## Data Availability

No new data were created or analyzed in this study. Data sharing is not applicable to this article.

## Supplementary Materials

The following supporting information can be downloaded at https://www.mdpi.com/article/10.3390/sports14090397/s1, Table S1: PRISMA 2020 Reporting Checklist and Manuscript Locations; Table S2: Database-Specific Search Strategies, Platforms and Update Notes; Table S3: QuADS Criteria and Complete Appraisal Matrix; Table S4: Component-Level Structured Narrative Confidence Assessment. The reporting, search, synthesis, confidence, and appraisal methods used in the supplementary materials correspond to the methodological sources cited in the main manuscript [15,16,17,18,19,20].
