# Supplementary material for: Training and Preparation Methods Relevant to Elite 5000 m Track Performance: A Systematic Review
Source: Sports (Basel). 2026 Sep 7;14(9):397. doi: 10.3390/sports14090397 (PMC13611246; doi:10.3390/sports14090397)
Supplement: Supplementary file 1 [file sports-14-00397-s001.zip › sports-4500071-supplementary.pdf]

Supplementary Materials

Training and Preparation Methods Relevant to Elite 5000 m Track Performance: A Systematic Review

Clinton Deon Swanepoel; Charl Jacobus Roux; Heather Morris-Eyton

These supplementary tables accompany the second-round revised manuscript. The historical February 2025 search syntax is transparently reconstructed from the archived search concepts because the exact original line-by-line database histories were not retained. The 17 August 2026 update search was prospectively documented.

Table S1. PRISMA 2020 Reporting Checklist and Manuscript Locations

Section/topic	Item	Requirement	Location
Title	1	Identify as systematic review.	Title.
Abstract	2	Summary consistent with review methods/results.	Abstract.
Rationale	3	Rationale in context of existing knowledge.	Introduction.
Objectives	4	State review question/objective.	Introduction.
Eligibility criteria	5	Specify inclusion/exclusion and grouping.	Sections 2.2 and 2.4; Tables 1-2.
Information sources	6	Specify databases and search/update dates.	Section 2.3; Table S2.
Search strategy	7	Present search strategies, fields, filters and limits.	Section 2.3; Table S2.
Selection process	8	Reviewer roles and dispute resolution.	Sections 2.5-2.6.
Data collection	9	Data extraction and reviewer roles.	Section 2.6; Table 4.
Data items	10	Outcomes and other variables.	Sections 2.2, 2.6; Table 4.
Risk of bias / quality	11	Appraisal tool and judgments.	Section 2.7; Table 3; Table S3.
Effect measures	12	Specify effect measures.	Narrative synthesis; quantitative results reported where available.
Synthesis methods	13	Grouping, synthesis and heterogeneity.	Section 2.6; Sections 3.3-3.7; Table 5.
Reporting bias	14	Assessment of reporting bias.	Section 2.8; Table S4.
Certainty/confidence	15	Assessment of certainty/confidence.	Section 2.9; Table 5; Table S4.
Study selection	16	Selection numbers/flow diagram.	Section 2.5; Figure 1; update search in Section 2.3.
Study characteristics	17	Characteristics of included publications.	Table 4.
Risk/quality results	18	Appraisal results per publication.	Table 3; Table S3.
Individual results	19	Summary findings per publication.	Table 4.
Synthesis results	20	Findings and limitations per synthesis.	Sections 3.3-3.7; Table 5.
Reporting bias results	21	Reporting-bias assessment.	Section 2.8; Table S4.
Confidence results	22	Confidence by component.	Section 3.7; Table 5; Table S4.
Discussion	23	Interpretation, limitations and implications.	Section 4.
Registration/protocol	24	Registration/protocol information.	Section 2.1; Section 4.2.
Support	25	Sources of support.	Funding statement.
Competing interests	26	Competing interests.	Conflicts of Interest statement.
Availability	27	Availability of data/materials.	Data Availability Statement; Supplementary Tables S1-S4.

Table S2. Database-Specific Search Strategies, Platforms and Update Notes

Historical search note: the original search was completed in February 2025. The exact original calendar day and exported line-by-line histories were not retained. The syntax below is therefore a transparent reconstruction from the archived search concepts. The same core concept blocks were retained for the 17 August 2026 update rather than retrospectively changing the terms to target particular publications. Because the original database histories and interface states were not retained, formal retrospective validation of the exact historical retrieval against every included publication cannot be claimed.

Database/platform	Search syntax	Fields/filters/truncation	Records retrieved
SPORTDiscus (EBSCOhost)	TX (("5000 meter track" OR "5000 m track" OR "middle distance running" OR "long distance track running") AND ("elite runner*" OR elite OR international OR "world class") AND (train* OR prepar* OR coach* OR pacing OR taper* OR altitude OR nutrition OR footwear))	TX/All Text; English; peer-reviewed; no original date restriction. * used as truncation.	Original n=701. Update 17 Aug 2026: n=5.
ProQuest Central	NOFT(("5000 meter track" OR "5000 m track" OR "middle distance running" OR "long distance track running") AND ("elite runner*" OR elite OR international OR "world class") AND (train* OR prepar* OR coach* OR pacing OR taper* OR altitude OR nutrition OR footwear))	Anywhere except full text (NOFT); scholarly/peer-reviewed; English; no original date restriction. * used as truncation.	Original n=521. Update 17 Aug 2026: n=0.
Scopus	TITLE-ABS-KEY(("5000 meter track" OR "5000 m track" OR "middle distance running" OR "long distance track running") AND ("elite runner*" OR elite OR international OR "world class") AND (train* OR prepar* OR coach* OR pacing OR taper* OR altitude OR nutrition OR footwear))	Title/abstract/keywords; article/review; English; no original date restriction. * used as truncation.	Original n=412. Update 17 Aug 2026: n=6.
PsycINFO (EBSCOhost)	TX (("5000 meter track" OR "5000 m track" OR "middle distance running" OR "long distance track running") AND ("elite runner*" OR elite OR international OR "world class") AND (train* OR prepar* OR coach* OR pacing OR taper* OR altitude OR nutrition OR footwear))	TX/All Text; English; peer-reviewed; no original date restriction. * used as truncation.	Original n=14. Update 17 Aug 2026: n=0.

Update-search flow: 11 records identified; 3 duplicates removed; 8 unique records screened; 0 additional eligible publications. The formal evidence set therefore remained 11 publications.

Table S3. QuADS Criteria and Complete Appraisal Matrix

Scoring: 0 = not reported/not met; 1 = limited; 2 = partially/adequately addressed; 3 = complete/detailed. QuADS is used here as a common methodological/reporting appraisal across diverse publication designs. The percentage categories are descriptive only and are not validated levels of methodological validity or formal design-specific risk-of-bias judgments.

1. Theoretical or conceptual underpinning

2. Statement of research aim(s)

3. Clear description of research setting and target population

4. Study design appropriate to stated aim(s)

5. Appropriate sampling

6. Rationale for data-collection tool(s)

7. Format/content of data-collection tool appropriate

8. Description of data-collection procedure

9. Recruitment data provided

10. Justification for analytic method

11. Analysis appropriate to research aim(s)

12. Research stakeholders considered

13. Strengths and limitations critically discussed

Study	1	2	3	4	5	6	7	8	9	10	11	12	13	Total	%	Descriptive category*
Alves et al. (2023)	2	3	3	3	2	2	3	3	1	2	3	1	2	30	76.9%	High
Aragon et al.	2	3	3	3	3	3	3	3	3	3	3	3	1	36	92.3%	High

Study	1	2	3	4	5	6	7	8	9	10	11	12	13	Total	%	Descriptive category*
(2016)																
Healey et al. (2022)	1	1	3	2	2	2	2	1	2	2	2	1	1	22	56.4%	Medium
Tjelta (2016)	2	2	2	3	3	2	3	3	2	3	2	2	0	29	74.4%	Medium
Jones and Whipp (2002)	0	0	2	1	2	0	1	2	2	1	2	0	0	13	33.3%	Low
Kenneally et al. (2021)	1	3	3	3	3	3	3	3	3	2	3	2	0	32	82.1%	High
Larsen and Sheel (2015)	1	2	2	2	3	2	2	3	3	2	2	3	1	28	71.8%	Medium
Sharma et al. (2017)	0	3	3	3	3	3	3	2	2	2	2	0	0	26	66.7%	Medium
Spilsbury et al. (2015)	1	3	3	3	2	2	3	2	2	1	2	2	1	27	69.2%	Medium
Stellingwerff et al. (2019)	2	3	3	3	3	2	2	3	2	3	3	1	1	31	79.5%	High
Thiel et al. (2012)	1	2	3	3	3	3	2	2	2	2	3	1	1	28	71.8%	Medium

Table S4. Component-Level Structured Narrative Confidence Assessment

Interpretation note: this is a structured narrative confidence judgment informed by GRADE concepts, not a formal outcome-specific GRADE assessment. The "initial evidence position" is a review-specific qualitative anchor based on publication design and evidence volume; it is not a formal GRADE starting level.

Component	Evidence basis	Initial evidence position	Methodological limitations	Inconsistency	Indirectness	Imprecision/evidence volume	Publication bias	Final confidence
Warm-up	1 randomized crossover 5000 m trial	Moderate anchor: experimental, single study	Generally good reporting; n=13	No between-study comparison possible	Event direct; caliber indirect (trained men, not Tier 4-5)	Single small study; no pooled estimate	Cannot assess formally	Low
Training-intensity distribution	1 world-class observational study + 1 review	Low anchor: observational + secondary	Mixed design; one review	Classification approaches differ	Elite but mixed events; not exclusive 5000 m	Small evidence volume; no pooled estimate	Cannot assess formally	Low
Tapering	1 elite survey/observational	Low anchor: observational, single study	Self-report/survey limitations	No replication within evidence set	Elite but mixed events; small 5000 m subgroup	Single study; small event-specific subgroup	Cannot assess formally	Very low
Altitude exposure/training responses	1 elite observational cohort	Low anchor: observational, single study	Observational; mixed intensities	No replication within evidence set	Elite 800-5000 m; training response at altitude, not 5000 m competition outcome	Single study; no event-specific effect estimate	Cannot assess formally	Very low
Pacing/tactical behaviour	2 elite observational + 1 conceptual	Low anchor: observational/conceptual	Observational; one conceptual source	Observational findings broadly consistent	Elite competition and 5000 m-inclusive; no causal training intervention	No pooled effect; limited causal evidence	Cannot assess formally	Low
Footwear technology	1 narrative/mechanistic review	Very low anchor: secondary, single source	Secondary evidence	No independent included comparison	Indirect to elite 5000 m outcome	Single secondary source	Cannot assess formally	Very low
Physiological profiling/performance determinants	1 narrative review	Very low anchor: secondary, single source	Secondary evidence	No independent included comparison	Broad elite distance-running evidence; not an intervention	Single secondary source	Cannot assess formally	Very low
Nutrition	1 narrative review	Very low anchor: secondary, single source	Secondary evidence	No independent included comparison	Broad middle-distance support evidence; no controlled elite 5000 m study	Single secondary source	Cannot assess formally	Very low
